# Nonbacterial Thrombotic Endocarditis With Leptomeningeal Carcinomatosis: A Rare Presentation of Advanced Rectal Adenocarcinoma

**DOI:** 10.7759/cureus.25713

**Published:** 2022-06-07

**Authors:** Polina Gaisinskaya, Michael Mamone, Kyle Kelschenbach

**Affiliations:** 1 Internal Medicine, Florida Atlantic University, Florida, USA

**Keywords:** nbte, libman sacks endocarditis, rectal adenocarcinoma, leptomeningeal carcinomatosis, nonbacterial thrombotic endocarditis

## Abstract

Nonbacterial thrombotic endocarditis (NBTE) is a rare condition characterized by the formation of sterile vegetations on valvular structures. Commonly asymptomatic in early stages resulting in systemic emboli as its initial manifestation. NBTE is found to be associated with diseases, which induce hypercoagulability, and, most commonly, is associated with malignancy. Rectal signet ring cell adenocarcinoma is an uncommon histological type of rectal cancer with a poor prognosis and aggressive nature. We present a rare manifestation of advanced rectal signet cell adenocarcinoma discovered due to manifestations of NBTE and leptomeningeal carcinomatosis.

## Introduction

Nonbacterial thrombotic endocarditis (NBTE), also known as verrucous endocarditis, Libman-Sacks endocarditis, or marantic endocarditis, is a rare condition characterized by the formation of sterile vegetations on valvular structures [1]. Since NBTE is asymptomatic in its early stages, the most common initial manifestation is a systemic embolism, often occurring as multiple emboli and subsequent multiple infarcts. NBTE is associated with conditions that confer a hypercoagulable state which include malignancy, systemic autoimmune diseases (i.e., systemic lupus erythematosus, antiphospholipid syndrome, systemic vasculitis), prothrombotic diseases (i.e., disseminated intravascular coagulation, protein S/C deficiency, Thrombotic Thrombocytopenic Purpura), and chronic inflammatory states (i.e., tuberculosis, chronic pyelonephritis, uncontrolled human immunodeficiency virus) [1]. Of these conditions, the highest association for NBTE is malignancy [2]. Rectal signet ring cell adenocarcinoma is an uncommon histological type of rectal cancer, at a rate of less than 1%, with a poor prognosis and aggressive nature [3]. A retrospective study of over 3,000 patients revealed a younger population (mean age 58), with a mortality rate of nearly half (44.1%) [4]. We present a rare manifestation of advanced rectal signet cell adenocarcinoma discovered due to manifestations of NBTE and leptomeningeal carcinomatosis.

## Case presentation

**Figure 1:**
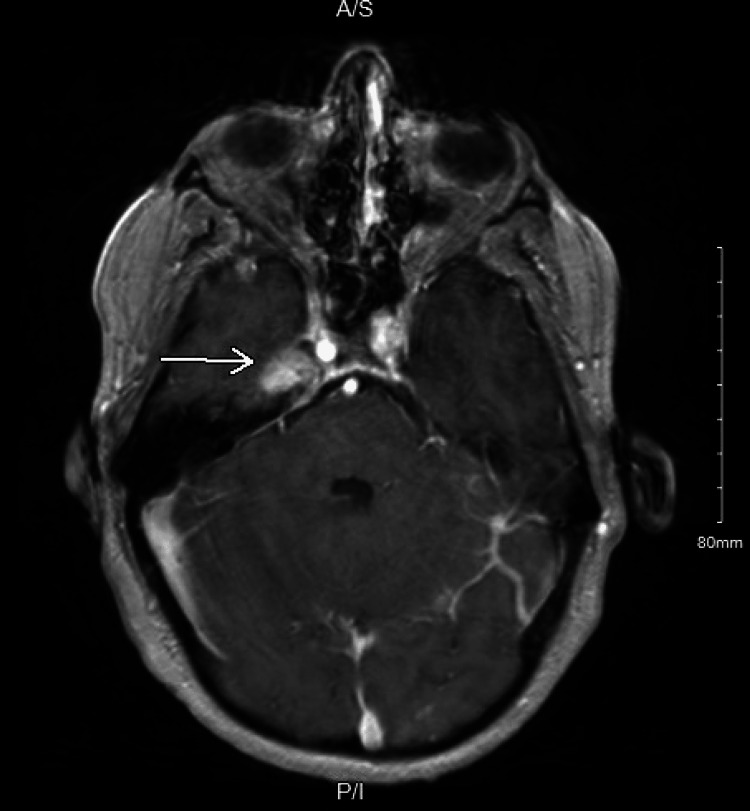
MRI brain revealing 1.4 cm mass at the posterior margin of Meckel’s cave (arrow)

A 48-year-old male with a past medical history of gangrenous appendicitis s/p appendectomy and recently diagnosed Bell’s palsy with concurrent trigeminal neuralgia presented to the hospital in November 2021 with chief complaints of bilateral lower extremity weakness and aphasia. He endured symptoms for two months up until the presentation. In September 2021, he experienced a right-sided facial droop with loss of sensation followed by bilateral lower extremity weakness in early November 2021. He reported a 20 lb weight loss, loss of appetite, severe fatigue, and bone pain in his bilateral lower extremities and back. He denied nausea, vomiting, and any changes in his stools. He denied undergoing a previous colonoscopy. He denied intravenous drug use. The patient delayed his presentation to the hospital as he was uninsured. On physical examination, the patient was tachycardia and had a right-sided facial droop, loss of sensation, and tongue deviation to the left. Bilateral lower extremities were ⅖ in muscle strength. Reflexes were intact. Initial labs revealed a white blood count (WBC) of 10.6 10^3^/uL, microcytic anemia, erythrocyte sedimentation rate (ESR) of 25 mm/h and alkaline phosphatase (ALP) of 379 U/L. MRI brain revealed multiple small scattered infarcts throughout both hemispheres and a 1.4 cm mass arising from the trigeminal ganglion on the right (Figure 1, arrow). 

**Figure 2:**
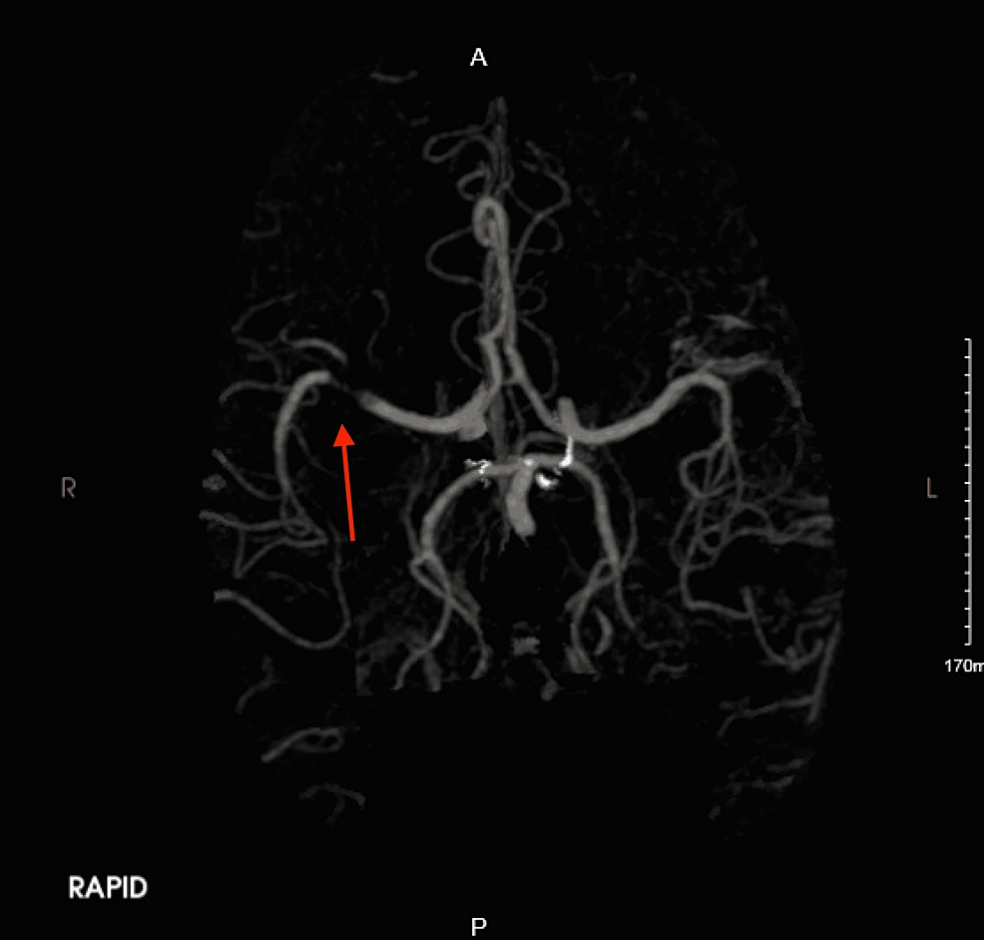
Head CTA revealed a 14 mL perfusion defect involving the left posterior frontal cortex (arrow)

Head CT angiography (CTA) revealed a 14 mL perfusion defect involving the left posterior frontal cortex (Figure 2, arrow)

**Figure 3:**
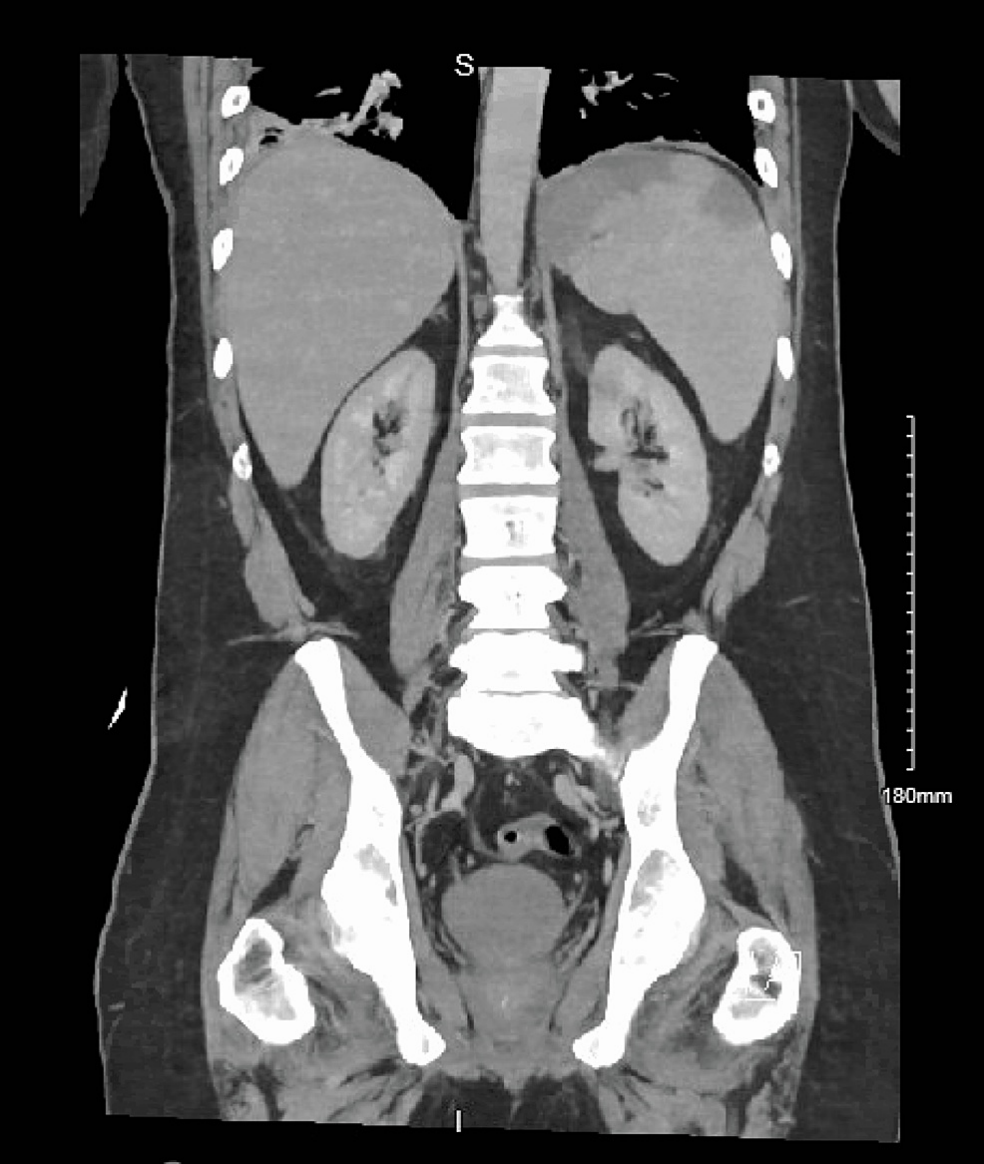
Several wedge-shaped splenic hypodensities are suspected infracts.

MRI L-spine revealed multiple sclerotic foci throughout the lumbar spine suspicious of metastatic disease. CT abdomen revealed a splenic infarct (Figure 3).

A transesophageal echocardiogram (TEE) showed a 1.1 x 1.2 x .5 cm mass on the anterior leaflet of the mitral valve. Three sets of blood cultures were obtained and set aside for Haemophilus species, Aggregatibacter species, Cardiobacterium hominis, Eikenella corrodens, and Kingella species (HACEK) organisms that were negative. Cerebrospinal (CSF) fluid was positive for elevated protein; however, was negative for malignant cells. The patient underwent a bone marrow biopsy of the right iliac, which showed metastatic poorly differentiated adenocarcinoma with mucinous and signet ring cell morphology. His colonoscopy showed a large friable mass in the rectum with biopsy-confirmed signet cell rectal adenocarcinoma. The patient was diagnosed with rectal adenocarcinoma with nonbacterial thrombotic endocarditis with presumptive leptomeningeal carcinomatosis. The hospital course was complicated by an acute right M1 occlusion, and he underwent a successful emergent thrombectomy. He had no residual defects. The patient underwent whole brain, skull base, and cauda equine radiation for leptomeningeal carcinomatosis with concurrent three cycles of folinic acid, fluorouracil, and oxaliplatin (FOLFOX) chemotherapy. 

## Discussion

Signet cell adenocarcinoma is rare and accounts for less than 1% of colorectal cancers. It is associated with a poor prognosis due to its aggressive nature. The symptoms of colorectal cancer typically involve a change in bowel habits, rectal bleeding, and iron deficiency anemia. This patient lacked the typical signs and symptoms associated with colorectal cancer and presented with those associated with NBTE due to systemic emboli, as well as signs and symptoms of leptomeningeal carcinomatosis. Literature review of the incidence of NBTE in the setting of signet cell rectal adenocarcinoma is limited due to its rarity [4]. A systemic embolism occurs more frequently in cases of NBTE secondary to malignancy, occurring in up to 50% of cases mainly involving the central nervous system. In patients with neoplastic disease and subsequent development of NBTE, the most common type of malignancy appears to be adenocarcinoma, with a high number of cases being of mucin-secreting and pancreatic origin. 

Given the similarity in presentation between NBTE and infective endocarditis, it is essential to rule out infections, including using select culture media and serology testing for both typical and atypical organisms. Since the clinical presentation of NBTE is nonspecific, its diagnosis relies on a high degree of clinical suspicion that can be complemented by imaging tests such as echocardiograms and transesophageal echocardiography to confirm the presence of valvular vegetations. A biopsy is not routinely recommended if infective endocarditis (IE) has been ruled out and suspicion for NBTE is high. Treatment is focused on the underlying condition; however, in the setting of malignancy, it is unknown whether NBTE improves with systemic chemotherapy. However, unlike IE and acute ischemic strokes, anticoagulation is recommended in NBTE. Current recommendations for systemic anticoagulation are with heparin [4]. Compared to heparin, direct oral anticoagulants were shown to be ineffective in the treatment of NBTE [4].

The patient also presented with signs of progressive trigeminal neuralgia and bell’s palsy which is believed to be attributed secondary to leptomeningeal carcinomatosis of the right trigeminal ganglion. Leptomeningeal carcinomatosis (LMC) is characterized by metastasis to the leptomeninges (arachnoid and pia maters) of malignant cells originating from a primary tumor. LMC occurs in approximately 5% of patients with cancer [4]. The most common primary tumors involved in LMC include breast (35%), lung (25%), lymphoma (11%), leukemia (8%), and melanoma (5%) [4]. Small cell lung carcinoma and non-small cell carcinoma represent 8% and 92% of the primary lung tumors involved in LMC, respectively [4]. There have been cases reported of LMC secondary to colorectal signet cell adenocarcinoma [5]. The most frequently affected cranial nerves in those with LMC are III, V, VI, VII, and VIII [4]. Patients with LMC will present with alteration in mental status, nausea, vomiting, and spinal symptoms such as weakness, paresthesias, and radicular pain. Positive CSF cytology is 100% specific for confirmation of LMC; however, malignant cells can be missed in the first sample in up to 40% of cases [4]. First negative cytology does not exclude the diagnosis of LMC. There is no consensus on the optimal medical management of LMC due to the low number of trials conducted. Due to inadequate trial data, case reports state that the prognosis of LMC is poor, and the treatment emphasizes symptomatic management and palliative care [6,7]. For symptomatic treatment, patients often receive opioids for analgesia, anticonvulsants for seizures, antidepressants, and anxiolytics. Patients with LMC also often undergo surgery, radiation, and chemotherapy. If left untreated, the median survival of patients with LMC is estimated to be 4-6 weeks [7]. 

## Conclusions

NBTE is asymptomatic in its early stages. If a patient presents with showering emboli, it is the most common initial manifestation with subsequent multiple infarcts, as seen in our case. NBTE is associated with conditions that confer a hypercoagulable state which includes malignancy, but rarely rectal adenocarcinoma. The major alternative diagnosis is infective endocarditis (IE), and negative cultures can facilitate the distinction between these two entities.

## References

[1] Busca C, Robles A, Ramos L (2019). Non-bacterial thrombotic endocarditis. Intech Open.

[2] Edoute Y, Haim N, Rinkevich D (1997). Cardiac valvular vegetations in cancer patients: a prospective echocardiographic study of 200 patients. Am J Med.

[3] Morales-Cruz M, Salgado-Nesme N, Trolle-Silva AM, Rodríguez-Quintero JH (2019). Signet ring cell carcinoma of the rectum: atypical metastatic presentation. BMJ Case Rep.

[4] Assi R, Hamieh L, Mukherji D, Haydar A, Temraz S, El-Dika I, Shamseddine A (2015). Leptomeningeal metastasis as initial manifestation of signet ring colorectal adenocarcinoma: a case report with review of literature. J Gastrointest Oncol.

[5] Bademci R, Bollo J, Martinez MC, Hernadez MP, Targarona EM (2019). Colorectal cancer prognosis: the impact of signet ring cell. Gastrointest Tumors.

[6] Ali S, Khan MT, Idrisov EA, Maqsood A, Asad-Ur-Rahman F, Abusaada K (2017). Signet cell in the brain: a case report of leptomeningeal carcinomatosis as the presenting feature of gastric signet cell cancer. Cureus.

[7] Le Rhun E, Preusser M, van den Bent M, Andratschke N, Weller M (2019). How we treat patients with leptomeningeal metastases. ESMO Open.

